# Burden of Caregivers of Patients with Chronic Diseases in Primary Health Care: A Cross-Sectional Study in Greece

**DOI:** 10.3390/nursrep14030122

**Published:** 2024-07-03

**Authors:** Eleni N. Albani, Aikaterini Toska, Constantinos Togas, Spyridon Rigatos, Viktor Vus, Evangelos C. Fradelos, Anastasios Tzenalis, Maria Saridi

**Affiliations:** 1 Department of Nursing, School of Health Rehabilitation Sciences, University of Patras, 26504 Patras, Greece; ealbani@upatras.gr (E.N.A.); s.rigatos@upatras.gr (S.R.); antzenalis@upatras.gr (A.T.); 2 Department of Nursing, School of Health Sciences, University of Thessaly, 41500 Larissa, Greece; atoska@uth.gr (A.T.); msaridi@uth.gr (M.S.); 3 Department of Psychology, Panteion University of Social and Political Sciences, 17671 Athens, Greece; togascostas@yahoo.gr; 4 Institute for Social and Political Psychology, National Academy of Educational Science of Ukraine, 04070 Kyiv, Ukraine; viktorvus@ukr.net

**Keywords:** caregivers, burden, anxiety, stress, primary health care, chronic diseases

## Abstract

Background: In the world of elderly people and people with chronic diseases, caregivers give a solution to caring at home. This study aimed to evaluate the burden of caregivers of patients with chronic diseases in primary health care and identify possible demographic and other determinants of it. Methods: This was a cross-sectional study with a convenience sample, which was conducted in two health centers. The sample comprised 291 caregivers who visited the aforementioned health centers in Patra, Greece. A composite questionnaire was utilized: the first part included demographic data and care-related information and the second included the Zarit Burden Interview and the Depression, Anxiety, and Stress Scale-21 (DASS-21). Results: The highest mean score in the DASS was recorded in the depression subscale and the lowest in the stress subscale. Concerning the Zarit Burden Interview, the highest mean score was recorded in the personal strain subscale and the lowest in the management of care subscale. The highest correlation was recorded between role strain and anxiety and the lowest was between management of care and stress. Similarly, the total score in the Zarit Burden Interview correlated significantly (in a positive direction) with depression, anxiety, and stress. Conclusions: Most of the caregivers of patients with chronic diseases in primary health care experienced a moderate to severe burden (especially in the dimension of personal strain) and moderate depression. The experienced burden was positively associated with depression, anxiety, and stress. There were significant differences in the caregivers’ burden according to several demographic and care-related characteristics.

## 1. Introduction

The increase in life expectancy and advancements in science have led to a situation where the global population is confronted with escalating chronic health issues. According to the World Health Organization (WHO), by 2050, 80% of older individuals will reside in low- and middle-income countries. Moreover, between 2015 and 2050, the proportion of the world’s population over 60 years old is projected to nearly double from 12% to 22% [1]. In Europe, over one-third (35.2%) of the population reported at least one chronic health problem in 2021 [2]. Additionally, individuals over 65 often experience limitations in daily activities, necessitating increased support from caregivers and resulting in financial burdens on either the health care system or the patients themselves [3].

Chronic diseases are defined broadly as conditions that persist for 1 year or more and necessitate ongoing medical attention, restrict activities of daily living, or both. The Centers for Disease Control and Prevention (CDC) categorizes the following conditions as chronic diseases: heart disease, stroke, cancer, type 2 diabetes, obesity, and arthritis [4].

Recognizing the imperative to mobilize the health care system to effectively manage the needs of the chronically ill and elderly populations promptly, the World Health Organization (WHO) has issued guidelines aimed at supporting caregiving by caregivers. These guidelines provide valuable recommendations and strategies to ensure that caregivers are adequately equipped to provide high-quality care and support to individuals with chronic health conditions and older adults, thereby enhancing their overall well-being and quality of life [5].

In this context, primary health care (PHC) plays a crucial role. PHC is defined as “essential health care” that is founded on scientifically sound and socially acceptable methods and technologies. It aims to make universal health care accessible to all individuals and families within a community. PHC initiatives facilitate the active involvement of community members in both implementation and decision-making processes. Services are delivered at a cost that is affordable for the community and the country at every stage of their development, fostering a sense of self-reliance and self-determination [6].

Informal care refers to the assistance and support provided by individuals who are not professional health care providers, typically involving family members or individuals from the broader social network [7]. In Greece, there is currently no specific legislation addressing informal caregivers, and generally, these caregivers do not receive support from institutional or social services. Many informal caregivers in Greece experience feelings of loneliness and lack adequate information and support, particularly due to the absence of formal home care services [8].

Related studies conducted in Greece have highlighted a notable increase in caregiver stress, particularly among female caregivers. Unfortunately, the country’s health system currently lacks institutional, social, and financial support for caregivers [9]. These studies underscore the significant burden placed on families caring for individuals with intellectual disabilities [10]. However, there is evidence to suggest that informal caregivers of patients with dementia who have participated in specialized training programs have experienced a significant reduction in mental burden. These informative training programs have been implemented in Greece in recent years, demonstrating their effectiveness in alleviating caregiver stress [11].

Additionally, caregiver age and the amount of time spent providing care to the patient are identified as exacerbating factors of caregiver burnout. Interestingly, research suggests that when the caregiver is the spouse of the patient, there is a lower likelihood of experiencing burnout compared to when the caregiver is a sibling [12].

In the Greek health system, there is a notable absence of public long-term care facilities specifically designed to support individuals with chronic diseases. As a result, informal caregivers often bear the primary responsibility for providing care to these individuals. The family structure in the country and its cultural roots increase the participation of typical caregivers in home care and general hospital care, which is true in Greece and other Mediterranean countries [13,14,15]. In many cases, patients with chronic diseases are transferred to hospitals for treatment, placing additional strain on both the patients and their caregivers. This seems to indicate a lack of recognition by the state regarding the contributions of informal caregivers. In 2016, caregivers and former caregivers created the Greek Carers Network EPIONI as a national non-profit organization. It aims to advocate for continuous and quality support services for individuals who work as informal, unpaid carers of family members or friends struggling with physical and/or mental illness, disability, or addiction [16].

The consequence of the lack of long-term care options is an increase in health care costs and operational challenges for hospitals. This occurs as beds in hospitals are occupied by patients who could potentially be supported in long-term care facilities or through home nursing services [17]. Addressing this issue will require a focus on strengthening self-care and self-management practices, as well as providing education to patients and caregivers. Additionally, integrating technology such as telemedicine and artificial intelligence will play a crucial role in enhancing the efficiency and effectiveness of health care systems in the future [18,19].

Carers of individuals with chronic health conditions represent a category of workers who deliver informal or formal health care and general health-related services. Unlike in Greece, where they lack institutional recognition as health care professionals [20], in the international context, carers are often officially acknowledged and recognized as health professionals. 

International studies demonstrate the interdependence between caregivers and the patients they support, as well as the impact that the patient’s level of functioning and course has on the caregiver’s mental health [21,22]. The duration of the care is also an important factor for the caregiver’s burden, i.e., the longer the care, the greater the caregiver’s burden and burnout [23].

In addition to the mental burden, caregivers frequently experience physical challenges arising from the tasks involved in patient care, as well as limitations in accessing medical equipment or utilizing telemedicine and homecare procedures [24,25].

Indeed, there is widespread agreement among international experts that primary health care provides the most suitable framework for designing, implementing, and maintaining comprehensive care and support services for caregivers. Within this framework, family doctors and nurses assume critical roles within multidisciplinary care teams, ensuring the delivery of holistic and well-coordinated care to both caregivers and their families [26,27].

This study aimed to assess the burden experienced by both formal and informal caregivers of patients with chronic diseases attending primary public health care centers. In Greece, there is a lack of systematic documentation regarding the needs of patients who receive informal care and the burden experienced by their caregivers. Additionally, the role of informal caregivers is not institutionally well established in Greece, and existing studies on caregiver groups are limited and fragmented [9]. Therefore, our study focuses on documenting a large cohort of caregivers who accompany patients to primary health care settings, aiming to investigate their needs and challenges. Based on the literature review, the following hypotheses were formulated:

**Hypothesis 1** **(H1).** *There is a significant correlation between caregivers’ burden and their levels of depression, anxiety, and stress.*

**Hypothesis 2** **(H2).** *There are significant differences in caregivers’ burden based on their demographic and care-related characteristics.*

**Hypothesis 3** **(H3).** *There are significant differences in caregivers’ levels of depression, anxiety, and stress.*

## 2. Methods

Study Design

This study employed a cross-sectional design with a convenience sample and was conducted at two health centers in Patras, Greece. The study duration spanned four months, from March to June 2023. Participants completed the questionnaires in the presence of the researcher.

Participants

They were eligible for inclusion if they met the following criteria:

➢ They were informal or formal caregivers of an individual diagnosed with at least one chronic physical or mental disease, such as cardiovascular disease, diabetes mellitus, Chronic Obstructive Pulmonary Disease, depression, dementia, etc.
➢ They were male or female individuals with sufficient ability to understand and respond to the questionnaire.
➢ They were aged 18 years or older.
➢ They could understand the Greek language.
➢ They voluntarily chose to participate in the research.

Caregivers who declined voluntary participation or were unable to respond to the questionnaire were excluded from this study.

Measures

A composite questionnaire was used in this study. The first part collected demographic data and care-related information, while the second part included the Zarit Burden Interview and the Depression, Anxiety, and Stress Scale-21 (DASS-21).

Demographic information

The caregivers provided information about their gender, age, marital status, place of residence, educational level, employment status, and whether they had children.

Care-related information

The caregivers provided information about the following aspects related to the patient: age, gender, chronic diseases, medications, their relationship to the patient (e.g., spouse, parent, etc.), cohabitation with the patient, hours/days devoted to caregiving, number of individuals living in the same house, total time spent caring for the patient, and whether they had another informal caregiver or a formal caregiver assisting them.

The Zarit Burden Interview

It is a widely used caregiver self-report measure consisting of 22 items, which is a revised version of the original 29-item questionnaire [28]. It assesses the sentiments of individuals caring for older people. Each item is a statement that caregivers rate on a 5-point scale, ranging from 0 (Never) to 4 (Nearly Always). The total score, obtained by summing item scores, ranges from 0 to 88, with higher scores indicating greater levels of caregiver burden. The burden is categorized into four dimensions: role strain, personal strain, relationship deprivation, and care management. Participants are classified based on their total score into categories such as little or no burden (0–20), mild to moderate burden (21–40), moderate to severe burden (41–60), and severe burden (61–88).

The factor structure of the Zarit Burden Interview has been subject to various interpretations by researchers, leading to different proposed models. In the Greek version utilized in this study, the scale comprises four factors as follows:

➢ Personal strain (9 items), which assesses the caregiver’s feelings of personal burden and loss of control over their life due to the care responsibilities. Example item: *“Do you feel that you have lost control of your life since your relative’s illness?”*
➢ Role strain (7 items), which examines the caregiver’s perception of being overwhelmed by the demands of caregiving and feeling that the care recipient asks for more assistance than necessary. Example item: *“Do you feel that your relative asks for more help than (s)he needs?”*
➢ Deprived relations (4 items), which evaluates the impact of caregiving on the caregiver’s social life and relationships outside of the caregiving role. Example item: *“Do you feel that your social life has suffered because you are caring for your relative?”*
➢ Management of care (2 items), which assesses the caregiver’s sense of responsibility and perceived adequacy in managing the care needs of the recipient. Example item: *“Do you feel that you should be doing more for your relative?”*

These factors provide a comprehensive assessment of caregiver burden, encompassing various dimensions of the caregiving experience as perceived by the caregiver [28].

The Zarit Burden Interview has been translated into multiple languages, including Greek, Chinese, French, Japanese, German, Hebrew, Spanish, Korean, Hindi, Portuguese, and more, demonstrating good psychometric properties across cultures. In this study, the Greek version of the scale was used [10], and Cronbach’s α coefficient was calculated to be 0.92 for the scale. For the subscales, Cronbach’s α was found to be 0.880 for “personal strain”, 0.804 for “role strain”, 0.744 for “deprived relations”, and 0.820 for “management of care”. These coefficients indicate high internal consistency reliability for each subscale, suggesting that the Greek version of the Zarit Burden Interview is a reliable measure for assessing caregiver burden in the Greek population.

Depression, Anxiety, and Stress Scale—21 Items (DASS-21)

It is a set of three self-report scales designed to measure emotional states related to depression, anxiety, and stress [29]. Each scale comprises 7 items, which capture various symptoms and experiences associated with these emotional states.

➢ Depression Scale: This scale assesses dysphoria, hopelessness, devaluation of life, self-deprecation, lack of interest/involvement, anhedonia (inability to experience pleasure), and inertia (lack of energy or motivation).
➢ Anxiety Scale: This scale evaluates autonomic arousal (e.g., heart palpitations, sweating), skeletal muscle effects (e.g., trembling, restlessness), situational anxiety (e.g., feeling tense or nervous in specific situations), and the subjective experience of anxious affect.
➢ Stress Scale: The Stress scale measures chronic nonspecific arousal, including difficulty relaxing, nervous arousal, being easily upset/agitated, irritability/over-reactivity, and impatience.

Respondents indicate the presence of symptoms over the previous week, with each item scored from 0 (indicating that the symptom did not apply at all over the past week) to 3 (indicating that the symptom applied to them very much or most of the time over the past week). Scores for depression, anxiety, and stress are calculated by summing the scores for the relevant items. Since the DASS-21 is a short-form version of the longer DASS-42, the final score for each item group needs to be multiplied by two (×2).

Table 1 typically presents the recommended cut-off scores for conventional severity labels for the Depression, Anxiety, and Stress Scale (DASS-21), helping to interpret the severity of symptoms reported by respondents.

In this study, the Greek version of the Depression, Anxiety, and Stress Scale (DASS-21) was utilized [30,31], and Cronbach’s α coefficients demonstrated adequate reliability for each subscale: depression (α = 0.767), anxiety (α = 0.787), and stress (α = 0.752). These coefficients indicate the internal consistency of the scale, suggesting that the items within each subscale are reliably measuring the intended constructs of depression, anxiety, and stress among the study participants.

Data analysis

The data analysis was conducted using SPSS version 28.0, with a significance level (*p*-value) set at 0.05. To assess the normality of continuous variables, the Kolmogorov–Smirnov test was employed. Descriptive statistics were used to summarize the data, while Pearson’s correlation coefficient was utilized to explore linear correlations among quantitative variables. Statistically significant differences in variables between two groups were examined using the *t*-test for independent samples, and differences among more than two groups were analyzed using ANOVA, with multiple comparisons conducted using the Bonferroni correction. Missing data were handled by excluding cases from analysis on a case-by-case basis.

Ethics

Approval for this study was obtained from the sixth Regional Health Authority of Peloponnese-Ionian Islands-Epirus and Western Greece (approval number 3689/13/03/2023). Caregivers were provided with detailed information about the study objectives and were assured of the anonymity and confidentiality of their responses. They were informed that their participation was voluntary and that they could withdraw from this study at any time without compromising the care provided to their patients. Additionally, participants were informed that their data would be used solely for research purposes. Written informed consent was obtained from all participants before their involvement in this study, and no remuneration was provided for their participation.

## 3. Results

The response rate for this study was 90.94%, with 291 out of 320 distributed questionnaires returned. The sample consisted of caregivers who visited the specified health centers. Demographic characteristics of both caregivers and patients, along with care-related information, are summarized in Table 2.

The caregivers reported caring for the patients for an average of 22.5 months (M = 22.52, SD = 32.01, Min = 1, Max = 240, Range = 239), with an average of about 8 h per day devoted to caregiving (M = 8.38, SD = 7.39, Min = 1, Max = 24, Range = 23). The most common medical conditions among the patients were cardiovascular diseases (13.4%), diabetes mellitus (9.6%), dementia (8.2%), and mobility disabilities (6.5%).

Additionally, it was found that men were more likely to have a formal caregiver than women, with a significant difference observed (χ2 = 17.616, *p* < 0.001).

Descriptive statistics for the subscales of the DASS and the Zarit Burden Interview are presented in Table 3. The depression subscale of the DASS had the highest mean score, while the stress subscale had the lowest mean score. For the Zarit Burden Interview, the personal strain subscale had the highest mean score, while the management of care subscale had the lowest mean score. It is important to note that due to differences in the number of items, direct comparison of mean scores between the subscales of the Zarit Burden Interview should be interpreted with caution.

Based on the total score in the Zarit Burden Interview, the participants were classified into the following burden categories:

- Little or no burden= 22 (10.6%);
- Mild to moderate burden= 34 (16.3%);
- Moderate to severe burden = 140 (67.3%);
- Severe burden= 12 (5.8%).

That is, the majority of them experienced a moderate to severe burden and a small percentage experienced a severe burden. 

The severity levels of depression, anxiety, and stress experienced by the caregivers (measured by the DASS-21 subscales) are presented in Table 4. 

Differences in the Zarit Burden Interview (total score) and in the DASS-21 across demographic and care-related characteristics are presented in Table 5. 

Concerning the Zarit Burden Interview, the following significant differences were found:

- Single participants had higher scores than married and separated/divorced (F = 19.684, df = 3189, *p* ≤ 0.001, ηp2 = 0.238).
- Those who did not cohabitate with the patients had higher scores than those who cohabitated (t = −2.829, *p* ≤ 0.007, Cohen’s d = −0.626).
- Those who did not have another informal caregiver also had higher scores than those who had such a caregiver (t = −2.646, *p* ≤ 0.011, Cohen’s d = −0.534) or a formal caregiver (t = 5.649, *p* ≤ 0.001, Cohen’s d = 1.026).

These findings suggest that marital status, cohabitation status, and the presence of other informal or formal caregivers may influence the perceived burden among caregivers, as assessed by the Zarit Burden Interview.

Concerning the DASS-21, only one significant difference was found: men had higher scores than women in anxiety (t = 2.059, *p* ≤ 0.001, Cohen’s d = 0.283). The total score in the Zarit Burden Interview was positively correlated with the caregiver’s age (r = 0.290, *p* < 0.001) and negatively with the months in care (r = −0.245, *p* < 0.001). The rest of the correlations are presented in Table 6. All of the Zarit Burden Interview subscales correlated significantly (in a positive direction) with the DASS-21 subscale. The highest correlation was recorded between role strain and anxiety and the lowest was between management of care and stress. Similarly, the total score in the Zarit Burden Interview correlated significantly (in a positive direction) with depression, anxiety, and stress. 

These results indicate that caregiver burden, as measured by the Zarit Burden Interview, is associated with various demographic and care-related factors, as well as with levels of depression, anxiety, and stress reported by caregivers.

## 4. Discussion

The objective of the present study was to evaluate the burden experienced by caregivers of patients with chronic diseases, utilizing a convenience sample from primary health care settings in Greece. This study was motivated by the recognition, supported by the existing literature, that caregiver burden is a significant global public health issue, associated with various health problems and psychological disorders among caregivers. 

In Greece, the lack of systematic recording of the participation of informal caregivers in patient care, as well as their needs, is an area worth investigating. The EPIONI program is an initiative aimed at supporting informal caregivers in Greece and promoting political planning to strengthen, train, and socially recognize informal caregivers [16]. By focusing on this issue within the Greek context, this study contributes to the broader understanding of caregiver burden and its implications for public health.

The present study’s main findings revealed that most caregivers experienced a moderate to severe burden, with a smaller percentage experiencing a severe burden. These results align with the existing literature, which emphasizes caregiver burden as a significant global public health concern associated with various health problems and psychological disorders [32]. The observed burden may be attributed to the substantial amount of time devoted to caregiving each day, approximately 8 h, as well as the demanding nature of certain chronic conditions, such as dementia and mobility disabilities, which necessitate intensive care. Indeed, the advanced age of most caregivers, averaging around 62 years, suggests that they may be experiencing chronic health conditions. This observation is supported by the positive correlation between the total score in the Zarit Burden Interview and the caregiver’s age. The prevalence of chronic illness underscores the need for individuals and caregivers to adapt and cope with these conditions in their daily lives. Developing innovative care models such as homecare, telecare, and caregiver education can be crucial in addressing the challenges posed by chronic illness and supporting both caregivers and care recipients effectively [33].

The negative association between the experienced burden and the duration of caregiving suggests that the initial months of caregiving are particularly challenging, potentially representing a “risky period” for higher burdens. However, as time progresses, caregivers and patients may develop effective adaptation strategies to manage the demands of the disease, leading to a reduction in perceived burden. This finding underscores the importance of providing support and resources to caregivers, especially during the early stages of caregiving, to help them cope with the initial challenges and develop effective coping mechanisms over time.

The caregivers’ burden documented in this study was higher than that recorded in another study conducted in Greece with a sample of chronic hemodialysis patients [34]. Consistent with the aforementioned study, the highest score was observed in the personal strain dimension.

Another significant finding is that all of the Zarit Burden Interview subscales correlated significantly (in a positive direction) with the DASS-21 subscale; that is, the higher the burden, the higher the depression, anxiety, and stress. The highest correlation was recorded between role strain and anxiety, and the lowest was between management of care and stress, and hypothesis 1 was confirmed.

The findings regarding the Zarit Burden Interview support hypothesis 2 to some extent. Specifically, single participants, caregivers who did not cohabitate with the patients, and those who did not have another informal caregiver had significantly higher burden scores compared to their counterparts.

Regarding the majority of our study sample, it appeared that male caregivers predominated. Although this finding could theoretically seem strange concerning the family structure in the country, it nevertheless seems to be a common finding in several studies from around the world, according to a recent meta-analysis [35]. Most of the caregivers in the study were family members, particularly a parent or the patient’s children. This result is in line with that found in other studies [34,36].

It is noteworthy that the vast majority of the caregivers did not cohabitate with the patients and had an additional formal caregiver. Those with this caregiving status experienced a higher burden than those who did not have a formal caregiver. This finding should be further evaluated in future studies. However, it could be justified by the fact that the need to manage serious illnesses and the special procedures that a chronically ill patient needs may be quite serious and not manageable by an informal non-health formal caregiver. Related studies show that patients with multimorbidities who receive long-term care from a caregiver show greater burnout and psychoemotional burden [37,38,39].

Gender has been examined in many studies, and female caregivers usually provide more immediate care and experience higher levels of burden and depression [40]. In this study, caregivers’ gender did not significantly affect their burden. The same result was found in another study in Greece [34]. On the other hand, men had higher scores than women in anxiety. Similar studies show that women caregivers including wives, daughters, and daughters-in-law tend to report worse health than caregivers who are men [40,41]. It has been suggested that there are several societal and cultural demands on women to adopt the role of a family caregiver [42]. Such gender differences were found consistently in both high-income and low- and middle-income countries [43,44].

Explanations of gender differences in caregiver burden may insist the unequal distribution of opportunities, and responsibilities may push women into the caregiver role more often than men and thus hamper their functioning in other fields (work, health) [45]. Also, the bibliography shows that the gender gap in the caregiver burden can also result from women and men dealing differently with the caregiving process, even if the conditions are similar [46]. Furthermore, concerning the DASS-21, only one significant difference was found, and men had higher scores than women in anxiety. That is, apart from this difference, hypothesis 3 was not confirmed.

This study acknowledges several limitations that may have affected the findings. Firstly, the cross-sectional design limits the ability to establish causal relationships between variables, and the absence of a longitudinal analysis restricts the understanding of temporal variations in the burden on caregivers. Secondly, convenience sampling can introduce bias into the results, making generalizations difficult. Thirdly, there is a potential for response bias, as participants may have provided socially desirable responses due to the nature of this study, which could affect the accuracy of the reported caregiver burden. Additionally, administering the questionnaires during visits to health centers may have introduced bias, as caregivers might have been influenced by the context of the health care setting and their emotional state at the time of completion. Lastly, this study did not assess the impact of income on caregiver burden, potentially underestimating important factors that influence the perceived burden experienced by caregivers.

### Practical and Clinical Implications

Despite the limitations mentioned above, the findings of the current research are valuable enough to offer recommendations for the effective management of the burden experienced by caregivers of patients with chronic diseases in primary health care. Health care professionals in primary care can assess caregivers’ issues, pinpoint vulnerable groups (considering variables that contribute to their burden), and refer them to other professionals, such as psychiatrists or psychologists. This approach will enable the provision of qualitative health care services.

It is recommended to conduct future research to delve deeper into and elucidate the results of the current study. One approach is to undertake a longitudinal study, aiming to overcome the limitations associated with a cross-sectional design. Additional investigation into various variables that showed no significant influence on caregivers’ burden in this study, such as patients’ gender or level of education, could provide further insights. Future studies might also concentrate on caregivers with specific characteristics or needs, such as those with minor children or those living alone. Lastly, researchers could explore the quality of the caregiver–patient relationship as a determinant of the caregiver’s experienced burden.

## 5. Conclusions

Most of the caregivers of patients with chronic diseases in primary health care experience a moderate to severe burden (especially in the dimension of personal strain) and moderate depression. The experienced burden is positively associated with depression, anxiety, and stress; that is, the higher the burden, the higher the depression, anxiety, and stress. There are significant differences in the caregivers’ burden according to several demographic and care-related characteristics. 

## Figures and Tables

**Table 1:** Dass-21 score severity levels.

Severity	Depression	Anxiety	Stress
Normal	**0–9**	**0–7**	**0–14**
Mild	**10–13**	**8–9**	**15–18**
Moderate	**14–20**	**10–14**	**19–25**
Severe	**21–27**	**15–19**	**26–33**
Extremely Severe	**28+**	**20+**	**34+**

**Table 2:** Caregivers’ and patients’ demographic characteristics and care-related information.

Caregivers’ Demographic Characteristics					
Age	Mean	SD	MIN	MAX	RANGE
	60.5	13.44	20	85	65
	**Frequency**		**Percentage%**		
**Gender**					
Man	**184**		**63.4**		
Woman	**106**		**36.6**		
**Residence**					
Village	**115**		**40.5**		
City	**14**		**4.9**		
Town	**155**		**54.6**		
**Job**					
Housewife	**28**		**9.9**		
Unemployed	**14**		**4.9**		
Civil servant	**71**		**25.0**		
Private employee	**52**		**18.3**		
Freelancer	**51**		**18.0**		
Pensioner	**68**		**23.9**		
**Educational level**					
Elementary school	**18**		**6.3**		
Secondary school	**59**		**20.8**		
Lyceum	**124**		**43.7**		
University/Technological Educational Institute	**66**		**23.2**		
M.A/M.Sc./Ph.D. Holder	**12**		**3.9**		
**Marital status**					
Single	**166**		**60.6**		
Married	**57**		**20.8**		
Separated/divorced	**21**		**7.7**		
Widow/er	**30**		**10.9**		
**Having children**					
Yes	**223**		**76.9**		
No	**67**		**23.1**		
**Relationship with the patient**					
Parent/child or child/parent	**173**		**67.6**		
Spouse/partner	**49**		**19.1**		
Brother/sister	**19**		**7.4**		
Other	**15**		**5.9**		
**Cohabitation with the patient**					
Yes	**58**		**20.5**		
No	**225**		**79.5**		
**Patients’ demographic characteristics and care-related information**					
**Age**	**Mean**	**SD**	**MIN**	**MAX**	**RANGE**
	**61.88**	**14.57**	**15**	**98**	**83**
	**Frequency**		**Percentage%**		
**Patient’s gender**					
Man	**75**		**27.3**		
Woman	**200**		**72.7**		
**Another informal caregiver**					
Yes	**51**		**18.5**		
No	**225**		**81.5**		
**Other informal caregiver (description)**					
Spouse					
Brother/sister	**15**		**29.41**		
Son/daughter	**12**		**23.52**		
Father/mother	**7**		**13.72**		
Children/friend	**6**		**11.76**		
**Formal caregiver**					
Yes	**194**		**69.8**		
No	**84**		**30.2**		
**Individuals living in the same house**					
1 individual	**29**		**10.0**		
2 individuals	**100**		**34.6**		
3–5 individuals	**148**		**51.2**		
6 or more individuals	**12**		**4.2**		
**Patients’ medication**					
Yes	**171**		**98.3**		
No	**3**		**1.7**		

**Table 3:** Descriptive statistics for the subscales of the Zarit Burden Interview and the DASS.

	N	Mean	SD	Min	Max	Range
**Zarit Burden Interview**						
**Personal Strain**	244	18.46	6.773	0	32	32
**Role Strain**	260	13.68	4.871	0	22	22
**Deprived Relations**	272	8.07	2.988	0	15	15
**Management of Care**	287	4.16	1.871	0	8	8
**Total Score**	238	43.88	15.149	0	69	69
**DASS-21**						
**Depression**	268	17.09	7.381	0	36	36
**Anxiety**	272	16.62	7.450	0	36	36
**Stress**	266	15.96	7.346	0	38	38

**Table 4:** Severity levels of depression, anxiety, and stress experienced by the caregivers (measured by the DASS-21 subscales).

Severity	Depression	Anxiety	Stress
Normal	37 (13.8%)	30 (11.0%)	122 (45.9%)
Mild	30 (11.2%)	10 (3.7%)	61 (22.9%)
Moderate	120 (44.8%)	65 (23.9%)	52 (19.5%)
Severe	60 (22.4%)	68 (25.0%)	27 (10.2%)
Extremely Severe	21 (7.8%)	99 (36.4%)	4 (1.5%)

**Table 5:** Differences in the Zarit Burden Interview (total score) and in the DASS-21 across demographic and care-related characteristics.

	Zarit Burden Interview	DASS-21		
		Depression	Anxiety	Stress
**Caregiver’s gender**				
Man	45.34	17.62	17.35	16.06
Woman	41.46	16.10	15.26	15.71
***p***	0.092	0.139	**0.041**	0.734
**Caregiver’s educational level**				
Elementary school	47.00	23.60	24.50	24.00
Secondary school	45.69	17.50	16.27	14.80
Lyceum	47.07	17.20	16.14	16.18
University/Technological Educational Institute	43.49	17.60	17.63	16.70
M.A/M.Sc. Holder	42.17	15.90	15.38	14.66
Ph.D. holder	35.75	15.00	13.83	13.45
***p***	0.374	0.216	0.065	0.054
**Caregiver’s marital status**				
Single	48.95	17.79	17.42	16.52
Married	32.10	15.16	15.21	14.98
Separated/divorced	32.27	14.95	13.40	14.32
Widow/er	41.90	18.36	17.07	16.38
***p***	**0** **.001**	0.062	0.056	0.421
**Having children**				
Yes	44.24	16.69	16.58	16.04
No	42.87	18.44	16.73	15.72
***p***	0.574	0.169	0.899	0.785
**Patient’s gender**				
Man	40.88	17.15	17.13	15.10
Woman	45.66	17.43	16.93	16.43
***p***	0.065	0.780	.846	0.200
**Cohabitation with the patient**				
Yes	36.94	16.78	16.75	16.71
No	45.78	17.39	16.74	15.82
***p***	**0.007**	0.646	0.991	0.552
**Another informal caregiver**				
Yes	38.08	15.36	15.74	15.35
No	45.69	17.76	16.90	16.14
***p***	**0.011**	0.120	0.459	0.576
**Formal caregiver**				
Yes	48.69	17.66	17.25	16.11
No	35.30	16.51	15.15	15.58
***p***	**0.001**	0.371	0.092	0.665

**Table 6:** Pearson correlations between the Zarit Burden Interview and the DASS-21 subscales.

	Depression	Anxiety	Stress
Personal Strain	0.440 **	0.441 **	0.344 **
Role Strain	0.455 **	0.482 **	0.357 **
Deprived Relations	0.386 **	0.421 **	0.366 **
Management of Care	0.222 **	0.283 **	0.179 **
Zarit Burden Interview—Total Score	0.493 **	0.519 **	0.399 **

*Note*: ** Correlation is significant at the 0.01 level (2-tailed).

## Data Availability

Data supporting this study are available from the corresponding authors upon reasonable request.

## Public Involvement Statement

No public involvement in any aspect of this research.

## Guidelines and Standards Statement

The reporting in the article adheres to the pertinent STROBE checklist for cross-sectional studies [29].
